# Cellulose-Based and Commercial Polyacrylate Hydrogels as Soil Amendments and Soilless Substrates for Microgreens Cultivation

**DOI:** 10.3390/gels12070611

**Published:** 2026-07-08

**Authors:** Aleksandra Mikhailidi, Lahbib Abenghal, Dan Belosinschi

**Affiliations:** 1 School of Medicine and Health Sciences, BAU International University Batumi, 237 Fridon Khalvashi Str., 6010 Batumi, Georgia; 2 Institut d’Innovations en Écomatériaux, Écoproduits et Écoénergies, Université du Québec à Trois-Rivières, 3351 Boul. des Forges, C.P. 500, Trois-Rivieres, QC G9A 5H7, Canada; lahbib.abenghal@uqtr.ca (L.A.); dan.belosinschi@uqtr.ca (D.B.); 3 Technologie Fiborizon, 360 Rue des Seigneurs, Saint-Etienne-des-Gres, QC G0X 2P0, Canada

**Keywords:** cellulose hydrogels, waste paper valorization, microgreens cultivation, soilless substrates, soil amendment, water retention, polyacrylate hydrogels, urban farming

## Abstract

Water scarcity restricts agricultural productivity and crop yield in many regions. Hydrogels have emerged as promising materials for soil amendment and soilless cultivation. Herein, cellulose hydrogels prepared from waste paper via a DMAc/LiCl solvent system (HG-WP) and from cotton microcrystalline cellulose via NaOH/aq. dissolution (HG-MCC) were investigated. HG-WP formed semi-solid, mechanically stable materials with high porosity, capable of retaining their shape during handling and plant cultivation, whereas HG-MCC appeared denser and less elastic. Their properties and agricultural performance were compared with those of a commercial polyacrylate hydrogel (HG-PA). The three hydrogels differed substantially in water capacity: HG-PA demonstrated the highest (50.13 g/g), whereas HG-WP and HG-MCC showed 30.57 and 3.66 g/g, respectively. Soil amended with 20 wt.% cellulose hydrogel exhibited improved water retention compared with untreated soil during the first 7 days of drying. Under laboratory conditions, hydrogels increased mustard biomass in soil under regular watering and plant survival under drought conditions. In soilless systems, cellulose hydrogels accelerated germination and improved plant development compared with control substrates. In soilless pea cultivation, HG-WP increased total biomass and survival from 56.7% to 79.3%. During basil cultivation in soil, cellulose hydrogel increased plant survival by 13.3% and mean biomass by 10.5% compared with the control. The results demonstrate the potential of cellulose hydrogels as biodegradable soil amendments and soilless substrates for short-cycle crop cultivation, outperforming the commercial polyacrylate hydrogel as an independent soilless substrate.

## 1. Introduction

Water scarcity is one of the major factors limiting the growth and productivity of agricultural crops. Climate change, accompanied by increasing temperatures and more frequent drought periods, reduces water availability and deteriorates soil properties, particularly in arid and semi-arid regions [1]. Under water stress conditions, seed germination, shoot and root growth, and overall crop productivity decrease significantly [2]. At the same time, the demand for more efficient water use is increasing both in industrial agriculture and in local cultivation systems, including urban farming. In recent years, technologies for cultivating microgreens and other short-cycle crops have become increasingly widespread, allowing plant production in confined spaces and within short cultivation periods [3,4]. Microgreens are characterized by rapid growth, high planting density, and shallow root systems, making substrate moisture stability critically important during the early stages of cultivation [5]. Because cultivation cycles usually last only 7–30 days, even short-term water scarcity can strongly affect germination, biomass accumulation, and seedling survival. In addition, soilless microgreen production systems require substrates capable not only of retaining water, but also of maintaining sufficient aeration and mechanical stability for root development [6].

One of the promising approaches to improving water-use efficiency is the application of hydrogels—polymeric materials capable of absorbing and retaining large amounts of water followed by its gradual release. Due to these properties, hydrogels reduce irrigation frequency, decrease water losses caused by evaporation and leaching, and maintain more stable conditions for plant growth [1,7,8,9]. According to reported studies, in some systems, hydrogels can provide plants with access to up to 95% of the retained water through gradual moisture release in the rhizosphere [10]. In addition, hydrogels can function as controlled-release systems for fertilizers and other active compounds, reducing nutrient losses and improving application efficiency [1]. Synthetic superabsorbent hydrogels based on polyacrylates are currently widely used in agriculture [11,12]. These materials are characterized by high water retention capacity, the ability to undergo multiple swelling cycles, and relatively low cost. However, their application is associated with several environmental limitations. Polyacrylate hydrogels exhibit poor biodegradability and may persist in soil for long periods, leading to accumulation of stable polymer residues [13]. Furthermore, synthetic superabsorbents are produced from petrochemical feedstocks, and possible toxic effects of their degradation products on plants and the environment have been discussed in the literature [9].

As a result, increasing attention is being paid to hydrogels based on natural polysaccharides, including cellulose, starch, alginate, and chitosan [7,9,14]. Such materials are biodegradable and can gradually decompose in soil under microbial activity, reducing the accumulation of persistent polymer waste [9,15]. In addition, polysaccharide hydrogels can improve soil structure, enhance water retention, and positively influence plant growth under limited moisture conditions [16,17]. Among natural polymers, cellulose attracts particular attention because of its abundance, low cost, and availability from diverse lignocellulosic feedstocks, including virgin biomass and recycled paper products. In particular, the use of waste paper as an alternative cellulose source represents a sustainable strategy that not only supports paper waste recycling but also enables the production of value-added functional materials for agricultural applications [18,19].

Existing approaches for preparing cellulose hydrogels include the use of both cellulose derivatives and non-derivatized cellulose [9,20,21,22,23]. The use of non-derivatized cellulose eliminates additional chemical modification steps, reducing cost and simplifying hydrogel preparation. Among existing approaches, DMAc/LiCl systems and aqueous NaOH solutions are of particular interest because they enable direct hydrogel preparation from cellulose without preliminary derivatization. The DMAc/LiCl system effectively disrupts intermolecular hydrogen bonding and enables formation of hydrogels with highly porous structures and high-water retention capacity. However, this method involves toxic and expensive solvents as well as difficulties associated with solvent regeneration and disposal [24]. Aqueous NaOH solutions or NaOH/urea systems are considered a more environmentally benign alternative, although cellulose dissolution efficiency in these systems is limited by cellulose crystallinity and the requirement for low temperatures to stabilize the solutions [19].

Despite the large number of studies devoted to hydrogel synthesis and structure, considerably less attention has been paid to hydrogel behavior under conditions close to real applications such as hydrogel drying kinetics, re-swelling ability, and degradation in soil, particularly for hydrogels prepared from secondary cellulose raw materials. Several studies have investigated cellulose-based hydrogels as soil amendments for conventional crops [13,17,19], and agarose hydrogels have been evaluated as soilless substrates for microgreen cultivation [6,25]; comparative studies of structurally distinct cellulose hydrogels prepared from secondary raw materials in microgreen cultivation systems remain scarce, and the potential of cellulose hydrogels from secondary raw materials as independent soilless cultivation substrates—an approach explored for the first time in the present work—has not been systematically addressed.

The aim of this work was to evaluate the potential of cellulose hydrogels prepared from non-derivatized cellulose for applications in urban farming technologies. The research question of this study was how the chemical nature of hydrogels and their mode of application (soil amendment or soilless substrate) influence water retention, hydrogel resistance to drying, and plant development during microgreen cultivation.

## 2. Results and Discussion

### 2.1. Hydrogel Properties

The cellulose source plays a crucial role in hydrogel preparation as well as in the selection of the most suitable production method. Various types of pulps can be used as cellulose sources, such as kraft pulp, sulfite pulp, thermomechanical pulp, and dissolving pulp, among others. Each type of pulp possesses distinct properties depending on its chemical composition, such as degree of polymerization, lignin content, ash content, and fiber morphology. All of these attributes can significantly influence hydrogel quality. For example, pulps with high lignin content are more difficult to swell and fibrillate because lignin is hydrophobic and increases fiber rigidity. Therefore, the raw materials used for hydrogel production, namely waste paper (WP) and microcrystalline cellulose (MCC), were first characterized in terms of lignin content, ash content, fiber length, and fiber width in order to identify the most suitable hydrogel preparation method. As shown in Table 1, the waste paper exhibited a relatively high lignin content (up to 14.2%) and high ash content (up to 9.3%). These results suggest the existence of thermomechanical pulp fibres and inorganic fillers respectively. The waste paper also has a long fiber length (1.6 mm), which highlights the need for a more efficient dissolution system such as DMAc/LiCl for hydrogel preparation. In contrast, MCC was much purer, containing only 0.5% lignin and 0.25% ash, with a fiber length not exceeding 0.13 mm. These characteristics explain the suitability of the NaOH dissolution method for MCC-based hydrogel production.

**Figure 1:**
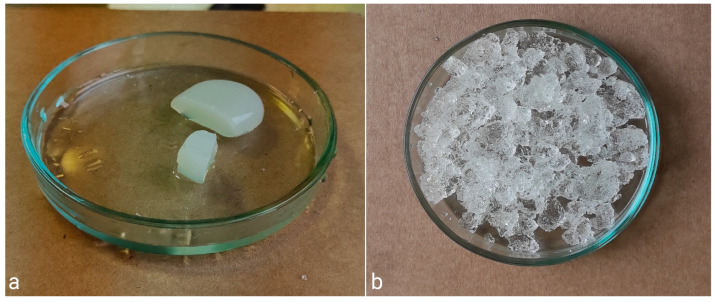
Representative appearance of cellulose hydrogel prepared from waste paper using the DMAc/LiCl method (**a**) and commercial polyacrylate hydrogel (**b**).

Cellulose hydrogels prepared from waste paper using the DMAc/LiCl method (HG-WP) formed semi-solid white to yellowish materials capable of retaining their shape during handling and prolonged storage in water (Figure 1a). The hydrogels exhibited limited elasticity and sufficient mechanical stability for plant cultivation experiments.

Structural and physicochemical properties of the similar WP-based hydrogels were described in our previous studies [18,26], where dissolution and regeneration were shown to induce transformation of cellulose I into cellulose II accompanied by partial reduction in crystallinity after regeneration and freeze-drying. Scanning electron microscopy (SEM) analysis of freeze-dried hydrogels revealed highly porous interconnected structures with pore sizes ranging from tens of nanometers to several micrometers depending on the waste paper source, while porosity values reached up to ~99%. Fourier-transform infrared (FTIR) spectroscopy and elemental analysis additionally demonstrated removal of non-cellulosic impurities originating from paper additives and printing components during dissolution and regeneration, resulting in higher chemical purity of regenerated hydrogels compared to the initial waste paper materials.

Hydrogel from microcrystalline cellulose (HG-MCC) prepared in NaOH medium formed materials visually similar to DMAc/LiCl-derived hydrogels, exhibiting grey-yellowish coloration, but appeared denser and less elastic. Similar differences between cellulose hydrogels prepared in DMAc/LiCl and NaOH-based systems were reported previously for regenerated cellulose films, where DMAc/LiCl-derived materials exhibited higher transparency and mechanical stability, while NaOH-treated systems produced less homogeneous and mechanically weaker structures [27]. These differences were attributed to variations in cellulose dissolution and structural rearrangement during regeneration.

The polyacrylate hydrogel (HG-PA) differed substantially in texture from cellulose-based hydrogels and behaved as a soft viscous gel mass rather than a monolithic material (Figure 1b). Such a structure is unsuitable for use as an independent soilless substrate because it lacks the mechanical stability and substrate density required to support root development and maintain the plant structure. In contrast, cellulose-based hydrogels retained a stable monolithic form (Figure 1a) and can therefore provide sufficient support for plant growth. This requirement for mechanical strength and substrate density in hydrogel-based cultivation systems has also been highlighted in the literature [1].

#### 2.1.1. Equilibrium Water Capacity

The investigated hydrogels demonstrated substantially different equilibrium water capacity (EWC) depending on polymer type, cellulose concentration, and dissolution method (Table 2).

HG-WP prepared via DMAc/LiCl dissolution demonstrated EWC of 30.57 g/g, which falls within the range reported for non-derivatized cellulose hydrogels (1.7–66.8 g/g) and is consistent with literature data for regenerated cellulose hydrogels of comparable origin [28]. Cellulose hydrogels obtained via DMAc/LiCl are not prone to re-swelling after drying due to hornification (discussed in Section 2.1.2).

HG-MCC prepared in NaOH/aq. Solvent system exhibited the lowest EWC (3.66 g/g) and partial re-swelling after drying. This is associated both with the higher cellulose concentration used during preparation (2.5 wt.%) and with structural differences characteristic of NaOH-based cellulose regeneration systems, which generally yield lower EWC compared to DMAc/LiCl-derived hydrogels [19,29,30].

HG-PA exhibited the highest EWC (50.13 g/g) and complete re-swelling after drying, typical for superabsorbent polyacrylate materials containing hydrophilic ionic groups. Although synthetic superabsorbent polymers are known to reach 400–600 g/g under laboratory conditions [31], the observed value is consistent with the known sensitivity of polyacrylate swelling to external factors such as ionic strength, pH, and temperature [32].

#### 2.1.2. Drying Kinetics in Air and Hornification of Cellulose Hydrogels

**Figure 2:**
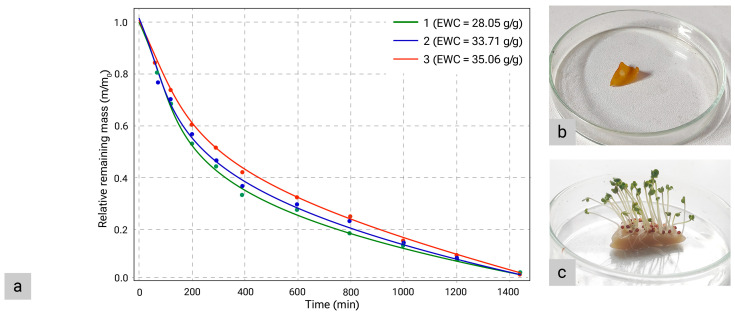
Drying behavior and structural changes in cellulose hydrogels. (**a**) Drying kinetics of cellulose hydrogels in air expressed as normalized remaining mass (m/m_0_), set to 1 for samples with different equilibrium water capacities: 1 (green) 28.05 g/g, 2 (blue) 33.71 g/g, 3 (red) 35.06 g/g; (**b**) hornification of cellulose hydrogel after drying under ambient conditions; (**c**) swollen cellulose hydrogel during mustard cultivation on day 3.

Drying kinetics of three HG-WP samples with different EWC values are presented in Figure 2a as normalized remaining mass (m/m_0_). The hydrogel with EWC of 28.05 ± 0.58 g/g (curve 1), 33.71 ± 1.05 g/g (curve 2), and 35.06 ± 1.16 g/g (curve 3) showed similar relative drying behavior during the initial period (0–120 min), with no statistically significant differences between any of the groups (ANOVA, Tukey test, *p* > 0.05). However, starting from t = 200 min, the sample with the highest EWC (curve 3) demonstrated statistically significantly slower relative mass loss compared to curve 1 (Tukey, *p* < 0.05), while curve 2 remained indistinguishable from both curves 1 and 3 throughout most of the experiment. By t = 390 min, curve 3 also differed significantly from curve 2 (*p* < 0.05). These results indicate that EWC influences relative drying rate primarily during the intermediate drying period (200–390 min), when capillary water retention becomes the dominant mechanism, whereas during the initial rapid evaporation phase the relative drying behavior is comparable across samples. By the end of 24 h, all hydrogels reached a dry state under ambient conditions.

The observed transition from comparable drying behavior in the initial period to diverging relative mass loss rates in the intermediate period is consistent with the two-stage drying mechanism described for regenerated cellulose gels, where an initial constant-rate period dominated by surface evaporation is followed by a falling-rate period controlled by internal capillary water transport [33].

Drying of cellulose hydrogels resulted in the formation of rigid non-swollen materials incapable of recovering their initial swelling capacity after rewetting. In cellulose science, this phenomenon is commonly referred to as hornification. The mechanism of cellulose hornification is associated with water removal via diffusion, leading to convergence of polymer chain surfaces and molecular contact between them [34]. This results in formation of irreversible interfibrillar contacts, intermolecular hydrogen bonds, and possible covalent cross-links that hinder water penetration during subsequent wetting [35]. As a result, the hydrogel loses its initial porous structure and swelling ability. The structural transformation is consistent with the nanoscale collapse of the porous cellulose network observed during drying of regenerated cellulose gel beads prepared via LiCl/DMAc, where water removal triggered irreversible aggregation of cellulose chains and formation of cellulose II crystalline domains [33].

Due to the rapid drying behavior of cellulose hydrogels in air, gauze layers were additionally used in some soilless cultivation experiments to support moisture retention within the substrate system. However, significant drying of hydrogel substrates containing plants was not observed during the cultivation period even in hydrogel-only systems. In control experiments performed without plants, cellulose hydrogels of the same size and shape as those used for mustard soilless cultivation were subjected to identical watering conditions. These control hydrogels gradually shrank and underwent hornification within 3 days (Figure 2b). In contrast, hydrogels containing growing mustard seedlings remained swollen throughout cultivation (Figure 2c). At the end of the experiment, hydrogels under dense planting conditions became thinner by approximately 48%, while their surface area decreased by 21%, indicating that drying still occurred but proceeded substantially more slowly than in hydrogels without plants.

The slowed drying of cellulose hydrogels in the presence of plants may, in our opinion, be associated with the formation of a localized humid microenvironment resulting from plant transpiration and hydrogel–root interactions. Root penetration into the hydrogel matrix may additionally mechanically limit hydrogel shrinkage during drying. Furthermore, plant coverage partially shields the hydrogel surface from direct air exposure, reducing evaporation rates. Together, these effects may lead to the formation of a self-regulating hydrogel–plant system capable of maintaining moisture for extended periods without rapid hornification.

To the best of our knowledge, such stabilization of cellulose hydrogel substrates by growing plants has not been previously described for soilless cultivation systems. The observed effect suggests that cellulose hydrogels may potentially function as independent substrates for cultivation of fast-growing plants with short vegetation periods.

#### 2.1.3. Soil Water Retention Capacity 

**Figure 3:**
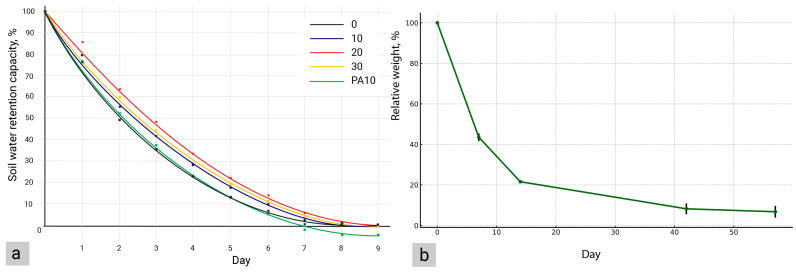
Water retention and mass change behavior of hydrogel-containing systems. (**a**) Water retention capacity for soil samples containing different mass concentrations of cellulose hydrogel HG-WP (10%, 20%, 30%), 10% polyacrylate hydrogel HG-PA, and control soil without hydrogel additives; (**b**) relative mass loss of cellulose hydrogel during exposure in soil over 57 days.

The soil water retention capacity for samples containing cellulose hydrogel at 10%, 20%, and 30% mass concentrations and 10% HG-PA, along with a control (0%), is shown in Figure 3a. HG-PA was tested at a single concentration of 10 wt.%, corresponding to the manufacturer’s recommended application rate and selected to minimize potential phytotoxic effects associated with higher polyacrylate concentrations [36]. The experiment was conducted over 11 days without additional watering.

Soil amended with 20 wt.% HG-WP showed the highest water retention, statistically significantly exceeding the control from day 1 to day 7 (ANOVA, Tukey test, *p* < 0.05). At 20 wt.%, optimal distribution within the soil matrix is likely achieved, enabling effective moisture retention. Other concentrations did not show statistically significant differences from the control (*p* > 0.05). A 10 wt.% concentration may be too low to form a water-retaining network, while at 30 wt.% the hydrogel may alter soil structure, compacting it and impeding water and oxygen circulation.

The drying kinetics curve for soil with polyacrylate hydrogel (Figure 3a, green line) coincided with the control until day 6, after which it dropped below the initial mass baseline—indicating that the polyacrylate hydrogel extracted water from the surrounding soil rather than releasing it. This is likely due to free sodium or potassium ions in the polyacrylate hydrogel creating an osmotic gradient, drawing water from soil (lower solute concentration) into the hydrogel (higher solute concentration). This behavior is fundamentally different from cellulose hydrogels, which release moisture to the environment.

However, since HG-PA has strong re-swelling capacity, even infrequent watering (1–2 times per 7 days) would allow it to restore its water content without extracting water from soil. By rapidly absorbing a substantial volume of water immediately after watering, HG-PA prevents water from draining to deeper soil layers, keeping it available for plants. Over time, it releases this water through evaporation. When evenly distributed, it can buffer moisture fluctuations throughout the soil matrix.

#### 2.1.4. Mass Loss of Cellulose Hydrogel During Soil Exposure

The relative mass of cellulose hydrogel samples decreased substantially during prolonged exposure in soil conditions (Figure 3b). The most pronounced mass loss was observed during the initial stage of the experiment, followed by a slower decrease over time. After 57 days, the residual mass of the hydrogel was below 10% of the initial value.

It should be noted that the observed mass loss may be associated with several concurrent processes, including water evaporation, partial structural destruction of the hydrogel, dissolution of soluble fractions, and possible biodegradation in soil [37,38]. The rapid initial mass loss is consistent with the evaporation of retained water and dissolution of loosely bound cellulose fractions, while the slower decrease observed in the later stages may reflect microbial degradation of the cellulose matrix. Biodegradation of cellulose in soil is mediated by cellulolytic microorganisms producing endoglucanases, exoglucanases, and β-glucosidases that collectively cleave β-1,4-glycosidic bonds, ultimately yielding glucose monomers [37]. The observed two-stage kinetics—rapid initial loss followed by a slower plateau—are qualitatively consistent with patterns reported for cellulose-based materials under soil burial conditions [38]. In addition, mechanical losses during sample extraction from soil and adhesion of soil particles to the hydrogel surface may have influenced the measured mass values. Therefore, the experiment should be considered as a preliminary assessment of hydrogel mass changes during soil exposure, indicating gradual destruction of the cellulose hydrogel under soil conditions.

### 2.2. Mustard Growth in Soil-Based Substrates

The experiment tested four soil substrates (Control, HG-WP, HG-MCC, HG-PA) under normal and water-stressed conditions.

#### 2.2.1. Shoot Height at Early Vegetative Stage

**Figure 4:**
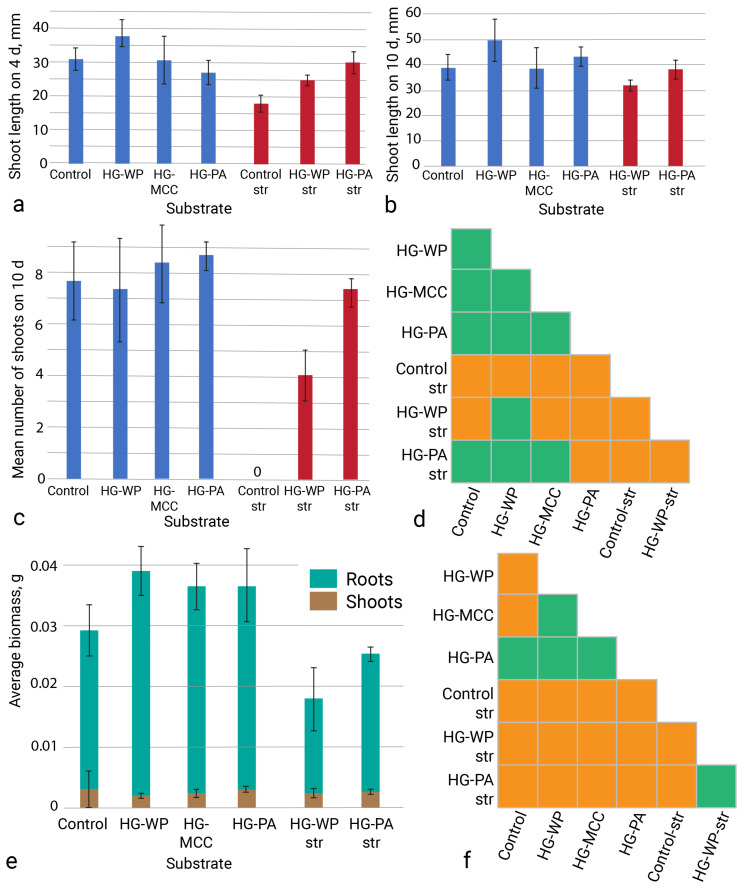
Growth parameters of mustard cultivated on soil-based substrates with and without hydrogel additives under regular and stress watering conditions. (**a**) Mean shoot height on day 4 under regular watering (blue bars) and water stress conditions (red bars); (**b**) mean shoot height on day 10; (**c**) mean number of shoots on day 10; (**d**) matrix of statistically significant differences in shoot number between substrate groups, Tukey tests (*p* < 0.05), orange and green cells indicate significant and non-significant differences, respectively; (**e**) mean biomass yield of mustard plants on day 10, including separately measured roots and shoots; (**f**) matrix of statistically significant differences in total biomass between substrate groups, Tukey tests (*p* < 0.05). Error bars represent standard deviation.

On day 4 of cultivation, the average shoot length of mustard seedlings was determined for all soil substrate groups under regular and stress watering conditions (Figure 4a). Statistical analysis revealed significant differences between groups (ANOVA, *p* < 0.05). According to Tukey’s post hoc test, no significant differences were observed between the control substrate and hydrogel-amended substrates under regular watering conditions despite substantial differences in EWC values of HG-WP, HG-MCC, and HG-PA (Table 2). Similarly, no statistically significant difference was detected between cellulose hydrogels HG-WP and HG-MCC, although their EWC values differed by more than one order of magnitude (Table 2). However, a significant difference was observed between HG-WP and HG-PA, with average shoot lengths of 37.8 ± 4.9 mm and 27.0 ± 3.5 mm, respectively.

Under water stress conditions, a different pattern was observed. All regularly watered groups differed significantly from the stress control group with reduced irrigation frequency (Figure 4a). In addition, a significant difference was detected between HG-WP and HG-WP-str, indicating that the type of irrigation regime influences plant development even in the presence of cellulose hydrogel additives. At the same time, no statistically significant difference was found between the regularly watered control and HG-WP-str groups, suggesting that cellulose hydrogel partially compensated for water scarcity during the initial stages of cultivation.

Within the stress treatment groups, a significant difference was observed between control-str and HG-PA-str. The synthetic HG-PA that is capable of efficient re-swelling apparently provided effective protection against water stress. This interpretation is supported by the similarity between shoot lengths observed for the regularly watered control substrate (30.9 ± 3.3 mm) and HG-PA-str (30.0 ± 3.2 mm).

#### 2.2.2. Number of Shoots and Survival at Harvest

By harvest, under regular watering conditions, no statistically significant differences in mean shoot number were observed between the control soil and hydrogel-amended substrates (HG-WP, HG-MCC, and HG-PA) according to Tukey’s post hoc test (*p* > 0.05) (Figure 4c,d). This indicates that incorporation of hydrogels into soil did not negatively affect seedling survival under sufficient irrigation conditions.

In contrast, all seedlings in the Control-str group died before harvest (by day 7), while hydrogel-containing substrates maintained partial plant viability under reduced watering frequency. Significant differences in shoot number were observed between the Control-str group and all regularly watered groups, as well as between Control-str and HG-PA-str (Figure 4d). Among stress-treated substrates, HG-PA-str demonstrated the highest shoot survival, whereas HG-WP-str retained a smaller number of viable plants.

At the same time, statistically significant differences were also observed between regularly watered hydrogel-containing substrates and their stress-treated counterparts, indicating that hydrogel addition alone could not fully compensate for prolonged water scarcity. Nevertheless, both HG-WP and HG-PA partially protected plants from complete mortality under drought conditions compared to the stress control without hydrogel additives.

#### 2.2.3. Biomass at Harvest

Biomass accumulation of mustard plants cultivated on hydrogel-amended soil substrates differed significantly between experimental groups (ANOVA, *p* < 0.05) (Figure 4e,f). Under regular watering conditions, both cellulose hydrogel substrates (HG-WP and HG-MCC) demonstrated significantly higher total biomass values compared to the control soil without hydrogel additives according to Tukey’s post hoc test. The increase in biomass was primarily associated with enhanced shoot development, while root biomass remained comparatively low in all groups.

The polyacrylate-amended substrate (HG-PA) also promoted higher biomass accumulation compared to the control. However, due to larger variability between replicates, the difference did not reach statistical significance (*p* > 0.05).

No statistically significant differences were detected between the hydrogel-containing substrates under regular watering conditions (Figure 4f), indicating that all investigated hydrogels similarly improved biomass production relative to untreated soil.

Under reduced watering frequency, biomass accumulation decreased substantially in all stress-treated groups. As described above, seedlings cultivated on soil without hydrogel additives under stress conditions did not survive until harvest. Nevertheless, both HG-WP-str and HG-PA-str maintained viable plants and measurable biomass production. Although the average biomass of plants grown on HG-PA-str (0.0255 ± 0.0015 g) exceeded that of HG-WP-str (0.0180 ± 0.0062 g), the difference between these substrates was not statistically significant because of the higher variability observed for HG-WP-str.

Pairwise comparison analysis (Figure 4f) additionally demonstrated statistically significant differences between most regularly watered and stress-treated groups, confirming the strong influence of irrigation regime on biomass accumulation. At the same time, the persistence of viable biomass in hydrogel-containing stress groups indicates that both cellulose and polyacrylate hydrogels partially mitigated the effects of water scarcity.

Overall, incorporation of hydrogels into soil substrates promoted increased biomass production under both normal and stress watering conditions, confirming their protective effect against moisture deficiency. Cellulose-based hydrogels demonstrated performance comparable to that of the synthetic polyacrylate hydrogel despite substantial differences in EWC values and re-swelling behavior.

#### 2.2.4. Shoot Length at Harvest

Interestingly, unlike biomass accumulation, shoot length at harvest (Figure 4b) demonstrated relatively small differences between most experimental groups. According to statistical analysis, no significant differences in shoot length were observed between the majority of substrates (*p* > 0.05), with the exception of HG-WP and HG-WP-str. This suggests that hydrogel amendments and irrigation regime influenced biomass accumulation more strongly than linear shoot growth. One possible explanation is that differences in plant water content and tissue density affected biomass values without substantially altering shoot elongation. The observed discrepancy between biomass and shoot length responses indicates that plant growth under hydrogel-containing substrates cannot be fully characterized by a single morphological parameter and requires comprehensive assessment of physiological and morphometric characteristics.

#### 2.2.5. Discussion

Previous studies have shown that cellulose-based hydrogels can enhance soil water retention and support plant growth under water-limited conditions [19,39]. The results obtained across all mustard cultivation experiments in soil further demonstrate that the effects of hydrogel amendments on plant development are determined not solely by water retention capacity but by a combination of physicochemical properties including structural characteristics, re-swelling behavior, and the chemical nature of the hydrogel. Both cellulose hydrogels (HG-WP and HG-MCC) promoted biomass accumulation comparably despite a nearly tenfold difference in EWC (30.57 vs. 3.66 g/g). This finding indicates that complete saturation water capacity may not be the most relevant parameter under cultivation conditions. Even limited additional water storage combined with gradual moisture release may contribute to improved water availability in the rhizosphere during short-term microgreen cultivation.

Although shoot length and survival did not differ significantly between regularly watered groups, both cellulose hydrogels significantly increased biomass accumulation relative to the control, indicating that hydrogel effects were expressed primarily through biomass production rather than seedling establishment.

Under regular irrigation, cellulose hydrogels provided plant growth promotion comparable to that of HG-PA despite their lower water retention capacity and substantially lower re-swelling capacity. Under intermittent drought, however, the superior re-swelling capacity of HG-PA became advantageous. Nevertheless, the comparable performance of cellulose hydrogels under regular watering highlights their potential as biodegradable alternatives to commercial polyacrylate hydrogels for short-cycle crop cultivation.

### 2.3. Mustard Growth in Soilless Substrates

#### 2.3.1. Germination Dynamics (2.5 seeds/cm^2^)

**Figure 5:**
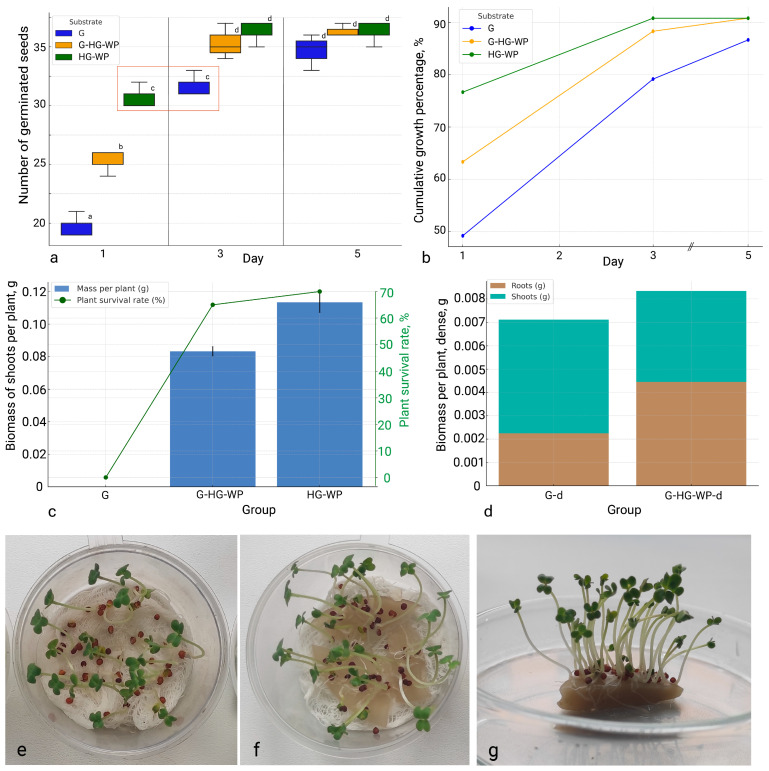
Productivity of mustard plants grown on different soilless substrates: G-control (gauze), G-HG-WP (cellulose hydrogel on gauze), and HG-WP (cellulose hydrogel). For (**a**–**c**), plants were grown with 40 seeds per pot (2.5 seeds/cm^2^), and for (**d**), plants were grown with a higher planting density (5 seeds/cm^2^). (**a**) Number of germinated seeds per day (different letters indicate statistically significant differences between groups, *p* < 0.05); (**b**) Cumulative germination percentage by day; (**c**) Average biomass of shoots per plant and survival rate (%) on the 10th day; (**d**) Biomass yield (mean of roots and shoots) per plant on the 10th day under high-density planting. Representative photographs of mustard plants cultivated on soilless substrates: (**e**) gauze substrate (G), (**f**) cellulose hydrogel on gauze substrate (G-HG-WP), and (**g**) cellulose hydrogel substrate (HG-WP).

Germinated seed counts on three soilless substrates (G, G-HG-WP, HG-WP) per day are shown in Figure 5a. Data in all groups were normally distributed (Shapiro–Wilk, *p* > 0.05 for all groups); variances were homogeneous (Levene’s test, *p* = 0.07). ANOVA and post hoc Tukey’s test revealed statistically significant differences between all substrate groups on day 1 (*p* < 0.001), but by day 3 significant differences were retained only between control and each hydrogel substrate (*p* < 0.05). By day 5, no statistically significant differences in germinated seed counts between the three substrate groups were detected (*p* > 0.05).

Control substrate (G) showed the slowest germination rate, starting at 49% on day 1 and reaching 87% on day 5 (Figure 5b). G-HG-WP reached 64% on day 1 and 91% by day 5. HG-WP showed the highest result on day 3 (77%) and also reached 91% by day 5. Notably, the number of germinated seeds on the control substrate reached the level observed on hydrogel substrate on day 1 only by day 3 (Figure 5a, red frame).

Thus, hydrogel substrate significantly accelerates mustard seed germination compared to the control in the early days of the experiment. By day 5, germination counts across three substrates converged, highlighting the effectiveness of hydrogels for a fast germination start.

#### 2.3.2. Biomass and Survival at Harvest (2.5 seeds/cm^2^)

By day 7, all plants in the control gauze substrate died, similarly to the control group cultivated in soil under water stress conditions, demonstrating that substrates without hydrogel components were unable to maintain sufficient moisture for mustard microgreen cultivation. On hydrogel substrates, high survival was observed: 65% for G-HG-WP and 70% for HG-WP (Figure 5c), and harvest was collected on day 10.

Mean shoot mass per plant on HG-WP was 0.0105–0.0119 g, exceeding the mass of G-HG-WP (0.0080–0.0087 g) (Figure 5c). The difference in plant mass between substrates was statistically significant (*t*-test, *p* < 0.05).

The inferior performance of the G-HG-WP substrate compared to HG-WP may be related to the capillary properties of gauze acting as a wick, drawing moisture from the hydrogel. Another possible reason is disruption of the microclimate around the roots: direct hydrogel–plant contact likely creates optimal conditions for growth (humidity, temperature), while gauze may disrupt this zone. These hypotheses require further investigation.

#### 2.3.3. High-Density Planting (5 seeds/cm^2^)

At higher planting density, shoot mass, root mass, and total biomass were measured on day 10. One of three replicates was excluded due to mold contamination, leaving two replicates per group, which may reduce statistical reliability.

Mean shoot mass per plant at G-HG-WP-d was approximately half of the same substrate with lower density (G-HG-WP): 0.0039 ± 0.0007 g vs. 0.0083 ± 0.0004 g. This indicates the negative effects of resource competition (water, light, nutrients) at higher densities (Figure 5d).

Comparison of high-density substrates showed that root mass per plant on G-HG-WP-d exceeded that on G-d. This confirms that hydrogel supports more active root system development even at an elevated planting density. Total plant biomass was also higher on the hydrogel substrate. Additionally, hydrogel substrate showed more uniform distribution of mass between roots and shoots, indicating harmonious plant development due to better moisture conditions.

#### 2.3.4. Discussion

The results of mustard cultivation in soilless substrates demonstrate that cellulose hydrogel HG-WP can function as an independent cultivation medium, providing sufficient moisture retention for seed germination and early plant development without soil support. The acceleration of germination on hydrogel-containing substrates (64% and 77% by day 1 for G-HG-WP and HG-WP, respectively, vs. 49% for gauze control) is consistent with the reported ability of cellulose hydrogels to enhance seed germination even without external water supply, attributed to sustained moisture availability at the seed–substrate interface [40].

The superior biomass performance of HG-WP compared to G-HG-WP suggests that direct hydrogel–root contact creates more favorable conditions for plant development. The highly porous interconnected structure of HG-WP (~99% porosity) likely facilitates both moisture delivery to roots and sufficient aeration of the root zone—two requirements identified as critical for hydrogel-based soilless substrates. The gauze layer in G-HG-WP may have acted as a capillary wick drawing moisture away from the hydrogel, while also disrupting the direct hydrogel–root interface and the localized humid microenvironment discussed in Section 2.1.2. At higher planting density, the reduction in biomass per plant is consistent with increased resource competition, while relatively better root development on hydrogel substrate compared to gauze control indicates that HG-WP maintains its moisture-buffering function even under elevated plant density.

Taken together, these findings suggest that non-derivatized cellulose hydrogels from waste paper represent a promising biodegradable alternative to conventional soilless substrates for short-cycle crop cultivation, with performance characteristics comparable to recently reported polysaccharide-based hydrogel systems achieving germination rates and fresh weights comparable to rockwool in full microgreen growth cycles [25,40].

### 2.4. Pea Growth in Soilless Substrates

Pea cultivation was conducted over 9 days with daily data collection. Three substrate types were used: gauze control (*n* = 3), cellulose hydrogel HG-WP (*n* = 4), and synthetic hydrogel HG-PA (*n* = 1). Since HG-PA had only one replicate, it was excluded from statistical analysis, but results are shown in figures for comparison.

#### 2.4.1. Germination Dynamics

**Figure 6:**
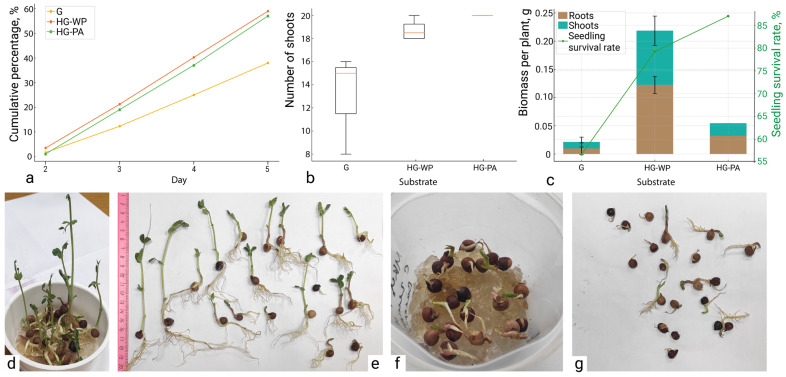
Germination dynamics, growth distribution, and biomass yield of pea plants cultivated on soilless substrates. (**a**) Cumulative percentage of germinated seeds over time; (**b**) distribution of shoot lengths on day 5 shown as median values with interquartile ranges; (**c**) shoot and root biomass per plant together with seedling survival rate on day 9. For HG-PA, standard deviation values are not shown due to *n* = 1. Representative photographs of pea plants cultivated on cellulose hydrogel substrate HG-WP (**d**,**e**) and polyacrylate hydrogel substrate HG-PA (**f**,**g**); (**e**,**g**) show all plants harvested from the corresponding cultivation containers.

The influence of hydrogel substrates on pea germination was evaluated by monitoring cumulative seed germination and shoot formation during the first 5 days of cultivation (Figure 6a,b).

Normality of germination data was verified using the Shapiro–Wilk test for G and HG-WP groups on days 2–5 (germination count stopped increasing after day 5). Data were normally distributed in most cases (*p* > 0.05), except on day 2 for both groups, likely due to variability in the initial stages of germination.

Given the predominantly normal distribution and homogeneity of variances (Levene’s test, *p* = 0.707), Student’s *t*-test was applied to compare G and HG-WP groups. The analysis revealed statistically significant differences between the substrates (t = −2.11, *p* = 0.045). The Kruskal–Wallis test, used as a non-parametric validation method, additionally confirmed the presence of significant differences (*p* = 0.022).

Cumulative germination curves (Figure 6a) demonstrated substantially faster germination on hydrogel-containing substrates compared to the gauze control. Both HG-WP and HG-PA groups showed steeper cumulative germination slopes, indicating accelerated seed activation and more intensive early seedling development in the presence of hydrogels. By day 4, cumulative germination on hydrogel substrates reached approximately 57–60%, whereas the control group remained below 40%.

The cellulose hydrogel HG-WP demonstrated the most stable germination behavior between replicates. As shown in the box plot (Figure 6b), HG-WP exhibited both the highest median shoot number and the smallest interquartile range, indicating not only improved germination efficiency but also greater reproducibility of seedling development. In contrast, the control group cultivated on gauze showed substantial variability between replicates, with shoot counts ranging from approximately 8 to 16 seedlings.

Interestingly, HG-PA demonstrated germination dynamics comparable to HG-WP despite major differences in hydrogel structure and mechanical properties. This result suggests that high water availability itself plays a dominant role during the early stages of pea germination, while differences in substrate rigidity become more important during subsequent seedling development.

Overall, the obtained results indicate that hydrogel-based substrates create more favorable moisture conditions for pea germination compared to gauze alone, leading to accelerated and more homogeneous seedling emergence.

#### 2.4.2. Biomass Yield

Biomass accumulation and seedling survival of pea plants cultivated on soilless substrates were evaluated on day 9 of cultivation. Visual inspection of the harvested plants additionally confirmed more intensive plant development and greater biomass accumulation for seedlings cultivated on the cellulose hydrogel substrate (Figure 6d–g). Shoot biomass, root biomass, and total seedling survival were determined for each experimental group (Figure 6c).

Shapiro–Wilk analysis confirmed normal distribution of biomass data for all measured parameters in the G and HG-WP groups (*p* > 0.05). Homogeneity of variances was additionally verified using Levene’s test (*p* > 0.05), allowing application of Student’s *t*-test for pairwise comparisons.

Statistical analysis revealed significant differences between the control and HG-WP substrates for all measured parameters. Pea plants cultivated on HG-WP showed higher shoot and root biomass together with increased seedling survival compared to the control. Root biomass represented the major contribution to total plant mass in both groups, indicating active root penetration and development inside the cellulose hydrogel matrix.

The poor performance of the G substrate after germination suggests that moisture retained by gauze alone was insufficient to support stable seedling development despite acceptable initial germination rates. In contrast, the cellulose hydrogel maintained a hydrated environment throughout cultivation, promoting continued biomass accumulation and higher plant survival. These results indicate that cellulose hydrogels can function as independent soilless substrates for cultivation of pea microgreens.

The HG-PA substrate demonstrated intermediate behavior. Biomass and survival values obtained for HG-PA were lower than for HG-WP but remained higher than for the control. Due to the availability of only one replicate for HG-PA, statistical analysis was not performed for this group. Nevertheless, the observed reduction in plant performance relative to HG-WP may indicate an inhibitory effect associated with prolonged direct contact between pea seedlings and the polyacrylate hydrogel substrate.

Unlike soil cultivation experiments, where hydrogel particles were dispersed within the substrate and plant contact with hydrogel was indirect, the soilless cultivation system involved continuous direct interaction between roots and the hydrogel matrix. Under these conditions, the weaker growth performance observed for HG-PA may reflect increased sensitivity of seedlings to the chemical composition or ionic environment of the synthetic hydrogel.

#### 2.4.3. Discussion

The results of pea cultivation in soilless substrates confirm that cellulose hydrogel HG-WP provides favorable conditions for both germination and subsequent seedling development, outperforming the gauze control across all measured parameters. The acceleration of germination on hydrogel substrates—with cumulative germination reaching ~57–60% by day 4 compared to below 40% on gauze—is consistent with the pattern observed for mustard (Section 2.3) and with the reported capacity of cellulose hydrogels to maintain continuous moisture availability sufficient for seed germination without frequent watering, as demonstrated for maize [41] and Choy Sum [40]. The higher reproducibility of germination on HG-WP, reflected in the smallest interquartile range among all groups, further indicates that the hydrogel substrate creates more homogeneous moisture conditions compared to gauze, where variability in water distribution between replicates was substantially higher.

The significantly higher shoot biomass, root biomass, and seedling survival on HG-WP compared to the gauze control demonstrate that cellulose hydrogel sustains plant development beyond the germination stage, maintaining a hydrated environment throughout the 9-day cultivation period. The dominant contribution of root biomass to total plant mass in the HG-WP group indicates active root penetration into the hydrogel matrix, suggesting that the porous cellulose network provides both physical support and moisture access for developing root systems. Hydrogel-based soilless substrates have been highlighted as a promising innovative practice for urban microgreen production [5], with porous architecture identified as critical for simultaneous water retention and root zone aeration [25].

The intermediate performance of HG-PA relative to HG-WP is consistent with the inhibitory tendency of polyacrylate hydrogels noted in soil cultivation experiments (Section 2.2), and may be amplified under soilless conditions where continuous direct root–hydrogel contact replaces the indirect particle-dispersed contact characteristic of soil amendment applications. Elevated Na^+^ concentrations associated with the polyacrylate manufacturing process may create osmotic stress at the root surface under prolonged direct contact [36,42], an effect that accumulates over the cultivation period but would not be apparent in short-term germination assays—consistent with the comparable germination dynamics observed for HG-WP and HG-PA despite diverging biomass outcomes. In contrast, the non-ionic nature of cellulose hydrogel eliminates such ionic effects, making HG-WP a more compatible substrate for prolonged direct root contact. To our knowledge, comparative evaluation of cellulose and polyacrylate hydrogels as independent soilless substrates for pea microgreen cultivation has not been previously reported, highlighting the novelty of these findings.

### 2.5. Basil Growth in Soil-Based Substrates

Basil cultivation experiments were performed over 30 days under regular watering conditions with irrigation every three days. Two substrate systems were compared: soil without additives (control) and soil amended with 20 wt.% cellulose hydrogel HG-WP.

**Figure 7:**
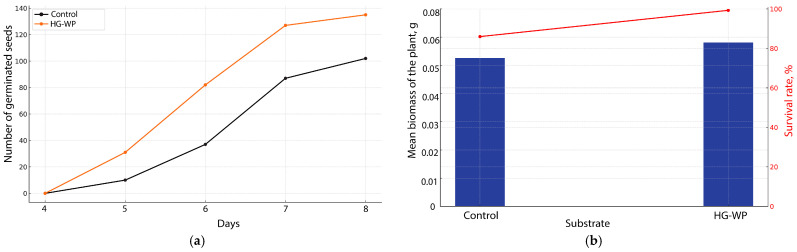
Effect of cellulose hydrogel HG-WP on basil cultivation in soil substrates. (**a**) Germination dynamics of basil seeds expressed as the number of germinated seedlings over time for control soil and soil amended with 20 wt.% HG-WP; (**b**) comparison of mean plant biomass per plant and seedling survival rate after 30 days of cultivation.

The addition of cellulose hydrogel positively influenced both seed germination dynamics and subsequent plant development (Figure 7a). During the first days after planting, germination proceeded substantially faster in the hydrogel-amended substrate. On day 5, the number of germinated seedlings in the HG-WP group was approximately three times higher than in the control substrate (31 and 10 seedlings, respectively). Although seedling numbers continued increasing in both groups during subsequent cultivation, the hydrogel-containing substrate maintained accelerated germination dynamics throughout the experiment. By day 8, 93.1% of all eventually germinated seeds had already emerged in HG-WP, whereas the corresponding value for the control substrate was 75.6%.

In addition to accelerated germination, hydrogel incorporation improved long-term plant survival and biomass accumulation (Figure 7b). After 30 days of cultivation, seedling survival in the hydrogel-amended substrate reached 99.3%, exceeding the control value (86.0%) by 13.3%. Mean biomass per plant was also higher in the HG-WP group, with an increase of approximately 10.5% relative to the control.

The observed effects are consistent with the water-retaining function of cellulose hydrogels in soil systems. Due to the relatively infrequent watering regime used in the basil experiment, the hydrogel likely acted as an intermediate water reservoir, reducing fluctuations in local soil moisture and creating more stable conditions for germination and plant growth over the extended cultivation period.

The accelerated germination dynamics observed in the HG-WP-amended substrate are consistent with the water-buffering function of cellulose hydrogels reported for other crops, where improved soil moisture stability was shown to promote earlier and more uniform seed emergence [40,43]. The observed 13.3% improvement in seedling survival and 10.5% increase in mean plant biomass after 30 days of cultivation align with the general trend reported in the literature, where hydrogel soil amendments improve plant-available water under infrequent irrigation regimes, leading to increased crop yield [44]. The relatively modest but consistent improvement in all growth parameters under regular watering conditions suggests that HG-WP acts primarily as a moisture buffer rather than a growth stimulant per se, reducing soil moisture fluctuations between irrigation events and maintaining more stable conditions for basil development over the extended cultivation period.

Notably, the positive effects of cellulose hydrogel were observed under a 30-day cultivation period with irrigation every three days—conditions substantially more demanding than the short-cycle microgreen experiments described in Section 2.2, Section 2.3 and Section 2.4. This indicates that the water-retaining function of HG-WP remains effective beyond the short-cycle application window despite the known hornification tendency of DMAc/LiCl-derived cellulose hydrogels upon drying, likely because the regular irrigation schedule prevented complete substrate dehydration throughout the experiment. These results extend the potential application range of waste paper-derived cellulose hydrogels beyond microgreen cultivation to longer-cycle herb production under moderate irrigation regimes.

## 3. Conclusions

Cellulose hydrogels prepared from waste paper and MCC via DMAc/LiCl and NaOH dissolution systems demonstrated promising potential as sustainable materials for the cultivation of short-cycle crops, while contributing to reduced water consumption through improved moisture retention and enhanced plant growth performance. Herein, the results demonstrate that cellulose hydrogels (HG-WP and HG-MCC) can effectively improve water retention, enhance seed germination, and promote plant growth under both soil and soilless cultivation conditions. Incorporation of 20 wt.% cellulose hydrogel significantly increased soil moisture retention during the first 7 days of drying compared to the control (*p* < 0.05). In basil cultivation, cellulose hydrogel addition increased plant survival by 13.3% and mean biomass by 10.5% after 30 days compared to the control substrate. Under soilless cultivation conditions, cellulose hydrogels also accelerated germination of mustard and pea and provided substantially higher pea biomass accumulation and seedling survival than the gauze control substrate. In contrast, the synthetic polyacrylate hydrogel (HG-PA) showed lower plant performance despite its high water capacity, indicating that hydrogel efficiency depends not only on water absorption capacity but also on structural properties, re-swelling behavior, and plant-hydrogel interactions. Additionally, cellulose hydrogels remained swollen throughout cultivation in the presence of growing plants, suggesting the formation of a localized humid microenvironment associated with root activity and transpiration. These findings highlight the potential of cellulose hydrogels as sustainable and biodegradable alternatives for agricultural applications, although further studies are needed to investigate their long-term degradation, structural properties, and root-hydrogel interactions under cultivation conditions.

Several limitations of this study should be noted. All experiments were conducted under controlled laboratory conditions, and the obtained results may not fully translate to field-scale applications where environmental variability is considerably higher. The hydrogels were evaluated exclusively for short-cycle crop cultivation, and their suitability for longer growing seasons requires further investigation. The hornification tendency of DMAc/LiCl-derived cellulose hydrogels upon drying reduces their re-swelling capacity, which limits their applicability in systems where substrates may dry between irrigation cycles. Additionally, the soil water retention experiments were performed using a single soil type, and results may differ for soils with varying texture, organic matter content, or ionic strength. Finally, the plant cultivation experiments covered three crop species, and performance of the developed hydrogels with other agriculturally relevant crops remains to be evaluated.

Future studies should focus on evaluating the performance of cellulose hydrogels under field conditions and with a broader range of agriculturally relevant crops. Investigation of root-hydrogel interactions at the microstructural level and the mechanisms underlying the observed humid microenvironment around growing roots would provide deeper insight into the biological effects of hydrogel substrates.

## 4. Materials and Methods

### 4.1. Materials

Microcrystalline cellulose derived from cotton raw material PH-102 (Vladimir Chemical Plant, Vladimir, Russia) and waste paper (MS-8B and MS-6B) were used as cellulose sources for hydrogel preparation. N,N-dimethylacetamide (DMAc, EKOS-1, Staraya Kupavna, Russia), anhydrous lithium chloride (LiCl, Komponent-Reaktiv, Moscow, Russia), ethanol (Vekton, Saint-Petersburg, Russia), sodium hydroxide (Oktanta, Saint-Petersburg, Russia), sulfuric acid (Vekton, Saint-Petersburg, Russia), and distilled water obtained by double distillation were used as solvents and auxiliary reagents. A commercial polyacrylate-based superabsorbent, supplied as a white powder mainly composed of potassium and sodium polyacrylate salts, was employed as a synthetic hydrogel for comparison.

Bleached cotton medical non-sterile gauze (39 g/m^2^, New Life, Navtex Cotton Mill, Navoloki, Russia) was used as a substrate for soilless cultivation. Ready-to-use nutrient soil for growing vegetables containing biohumus and agroperlite, pH ≥ 5.8 (Terra Vita, Nevatorph, NORD PULP LLC, Fornosovo, Russia), was used as the soil substrate. Seeds of leaf mustard Ladushka (*Brassica juncea* L.), garden sugar pea Karubi (*Pisum sativum* L.), and basil Orion (*Ocimum basilicum* L.) were obtained from Sortsemovoshch, Saint-Petersburg, Russia. Seed disinfection and planting were performed according to the manufacturer’s recommendations provided on the packaging. All chemicals were of analytical or chemically pure grade and were used without further purification.

### 4.2. Hydrogel Preparation Methods

#### 4.2.1. Cellulose Hydrogels from Waste Paper (DMAc/LiCl Method)

DMAc/LiCl solvent system was applied to prepare cellulose hydrogels from waste paper according to a previously described procedure [18]. Briefly, waste paper was subjected to thermomechanical defibration in water, followed by solvent exchange (water–ethanol–DMAc) and dissolution in DMAc containing 7 wt.% LiCl (DMAc/LiCl) under stirring at 70 °C for 3–5 h. Undissolved residues were removed by filtration or centrifugation (the latter being preferred due to the high viscosity of the solutions). The insoluble fraction was washed with water, dried at 105 °C to constant mass, and weighed. The actual cellulose concentration of each solution was calculated based on the dissolved fraction only. The obtained cellulose solutions (1.0 wt.%) were cast into molds and kept under ambient conditions for 2–4 days until organogels formed. Hydrogels were obtained by repeated rinsing of the organogels with water to remove residual DMAc/LiCl. The cellulose hydrogels prepared using this method were designated as HG-WP in this study. The resulting hydrogels remained stable during prolonged storage in water at ambient temperature without noticeable structural degradation.

#### 4.2.2. Cellulose Hydrogels from MCC (NaOH Method)

NaOH-based method was used to prepare cellulose hydrogels from MCC using a freeze–thaw process adapted from Tovar-Carrillo et al., with slight modifications [27]. Briefly, MCC was preliminarily treated with 15 wt.% sulfuric acid and washed with water to neutral pH. The pretreated cellulose was dispersed in 9 wt.% NaOH solution to obtain a cellulose consistency of 2.5 wt.% and stirred for 1 h at room temperature until a homogeneous dispersion was obtained. The system was then frozen at −15 °C for 12 h and thawed at ambient temperature to induce gel formation. The obtained hydrogels were rinsed with 10 wt.% sulfuric acid and subsequently rinsed with water until neutral pH was reached. The swollen hydrogels were stored in water at ambient temperature until further use, and the samples prepared using this method were designated HG-MCC in this study.

#### 4.2.3. Polyacrylate-Based Hydrogel

A PA hydrogel was prepared by adding distilled water to the commercial superabsorbent polyacrylate powder material to obtain a final polymer concentration of 1.5 wt.%. Upon water addition, rapid swelling occurred, resulting in immediate gel formation. The resulting hydrogel was stored in a sealed container at ambient temperature until further use, and the hydrogel prepared by this method was designated HG-PA in this study.

### 4.3. Characterization of Hydrogels

#### 4.3.1. Ash Content

The ash content of the waste paper, MCC, and the hydrogels produced from these materials was evaluated following the ISO 2144 standard procedure [45]. Prior to analysis, porcelain crucibles were cleaned, dried, labeled, and weighed using an analytical balance. About 1.5–3.5 g of oven-dried sample was then added to each crucible, followed by a second weighing. All measurements were carried out in triplicate to ensure reproducibility. The loaded crucibles were heated in a muffle furnace at 900 ± 25 °C for 24 h to remove the organic fraction. After calcination, the crucibles were transferred to a desiccator, allowed to cool to room temperature, and weighed to determine the amount of remaining inorganic residue. The ash content was subsequently calculated using the following equation:

(1) $$\mathrm{Ash}\;(\%)=\frac{\left(m_{2}-m_{0}\right)}{\left(m_{1}-m_{0}\right)}\times 100,$$

where *m*_0_ is the mass of the empty crucible, *m*_1_ is the mass of the crucible containing the dry sample, and *m*_2_ is the mass of the crucible containing the ash.

#### 4.3.2. Lignin Content

The lignin content of each sample was determined following the TAPPI T 222 standard method [46]. Briefly, approximately 1.5–2 g of oven-dried material was mixed with 72% sulfuric acid and maintained at room temperature for 2 h to hydrolyze the carbohydrate fraction. The acid concentration was subsequently reduced to 3% by dilution, and the suspension was heated under reflux at 100 °C for 4 h to complete the hydrolysis process. After treatment, the mixture was filtered, and the remaining insoluble lignin residue was dried at 105 ± 3 °C for 24 h. The lignin percentage was then determined using the following equation:

(2) $$\mathrm{Lignin}\;(\%)=\frac{m_{residue}}{m_{sample}}\times 100,$$

#### 4.3.3. Fiber Length Distribution

The fiber length distribution of the samples investigated in this study was analyzed using a fiber quality analyzer (L&W Fiber Tester Plus, Lorentzen & Wettre, Sweden). The arithmetic mean fiber length and fiber width were measured to assess the influence of the reagents and processing conditions on fiber sizes.

#### 4.3.4. Equilibrium Water Capacity and Re-Swelling

Equilibrium water capacity was determined gravimetrically by measuring the masses of hydrogels in swollen and dried states [41]. Drying was carried out under ambient conditions until constant weight was achieved. *EWC* (grams of water per gram of dry hydrogel, g/g) was calculated according to Equation (3)

(3) $$EWC\;(g/g)=\frac{m_{sw}-m_{dry}}{m_{dry}},$$

where *m*_sw_ is the mass of the swollen hydrogel (g), and *m*_dry_ is the mass of the dried hydrogel (g).

Re-swelling behavior was evaluated by immersing previously dried hydrogel samples in 40 mL of distilled water for 24 h, followed by weighing and subsequent drying under ambient conditions until constant weight. *EWC* after re-swelling was calculated using Equation (3). Re-swelling efficiency (*R*, %) was calculated according to Equation (4)

(4) $$R\left(\%\right)=\frac{{EWC}_{2}}{{EWC}_{1}}\cdot 100,$$

where *EWC*_1_ is the initial water retention capacity of the hydrogel and *EWC*_2_ is the water retention capacity after re-swelling.

#### 4.3.5. Drying Kinetics of the Hydrogels in Air

Drying kinetics of cellulose hydrogels in air were studied gravimetrically. Weighted hydrogel samples were placed in open containers at 20 °C and 45% relative humidity. Sample mass was recorded at regular time intervals until constant weight was reached.

#### 4.3.6. Soil Water Retention Capacity 

Soil water retention capacity was evaluated gravimetrically according to a previously reported approach for hydrogel-amended soils [47]. Soil samples containing swollen cellulose hydrogel HG-WP (10, 20, and 30 wt.% relative to dry soil mass) and polyacrylate hydrogel HG-PA (10 wt.% relative to dry soil mass) were investigated. Soil without hydrogel was used as the control. The samples were saturated with water and kept in open containers under ambient laboratory conditions without additional watering for 11 days to evaluate natural drying kinetics. Sample mass was recorded daily until stabilization of weight loss.

#### 4.3.7. Mass Loss of Hydrogels During Soil Exposure

Changes in hydrogel mass during soil exposure were evaluated gravimetrically. Hydrogel samples were fixed on thin wire holders and placed in soil under ambient laboratory conditions. The samples were periodically removed for weighing over a 57-day period. Before weighing, residual soil particles were carefully removed from the hydrogel surface using a soft brush. The relative weight (%) of the samples was calculated as a percentage of the initial mass according to Equation (5)

(5) $$Relative\;Weight\;\left(\%\right)=\frac{Current\;Weight}{Initial\;Weight}\cdot 100,$$

The measurements were used to evaluate the overall mass change in the hydrogels during prolonged exposure in soil.

#### 4.3.8. Experimental Design for Plant Cultivation in Hydrogel Substrates

**Figure 8:**
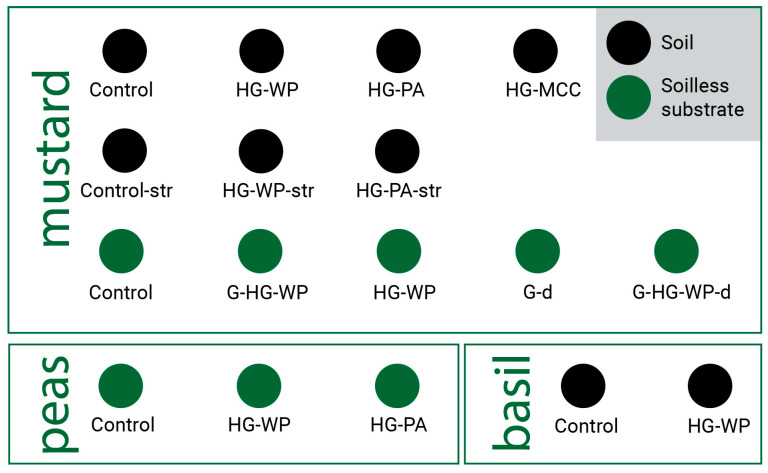
Experimental design for the cultivation of mustard, pea, and basil in soil-based and soilless substrates. Black circles represent soil cultivation, while green circles represent soilless cultivation. Substrates: Control (for soil-based substrate)—soil without additives; Control-str—soil without additives under water stress conditions; HG-WP—soil supplemented with cellulose hydrogel prepared using the DMAc/LiCl solvent system; HG-WP-str—soil supplemented with cellulose hydrogel prepared using the DMAc/LiCl solvent system under water stress conditions; HG-PA—soil supplemented with polyacrylate hydrogel; HG-PA-str—soil supplemented with polyacrylate hydrogel under water stress conditions; HG-MCC—soil supplemented with cellulose hydrogel prepared using the NaOH solvent system; Control (soilless cultivation)—gauze; G-HG-WP—cellulose hydrogel prepared using the DMAc/LiCl solvent system placed on gauze; HG-WP—cellulose hydrogel prepared using the DMAc/LiCl solvent system; HG-PA—polyacrylate hydrogel; G-d—gauze under high-density seed planting conditions; G-HG-WP-d—cellulose hydrogel prepared using the DMAc/LiCl solvent system placed on gauze under high-density seed planting conditions.

The experimental design for cultivation of mustard, pea, and basil in soil-based and soilless hydrogel substrates is presented in Figure 8. For soil cultivation experiments, swollen hydrogels were mixed with the soil substrate before seed planting. Cellulose hydrogel additives prepared using DMAc/LiCl (HG-WP) and NaOH (HG-MCC), as well as polyacrylate hydrogel (HG-PA), were investigated. Soil without hydrogel addition was used as the control. Mustard and basil cultivated in soil substrates under regular watering conditions were investigated. Additional water stress experiments with irregular watering were carried out for mustard cultivation.

For soilless cultivation experiments, seeds were placed on gauze (G), directly on hydrogel substrates (HG-WP), or on combined gauze–hydrogel substrates (G-HG-WP). G was used as the control substrate. Soilless cultivation experiments were performed under regular watering conditions. Additional experimental series included high-density seed planting in order to evaluate the effect of hydrogels under increased competition between plants.

The experiments were carried out under ambient laboratory conditions. Plant growth and substrate conditions were visually monitored throughout the cultivation period. Each experimental series was performed in triplicate.

### 4.4. Soil-Based Substrate Preparation

Before planting, soil was disinfected by a single watering with a dilute potassium permanganate solution. Based on results from a preliminary experiment determining soil water retention capacity, swollen hydrogel was added at 20 wt.% of the total soil mass per pot.

### 4.5. Seeds and Planting

Pea seeds were rinsed in cold water, treated with 0.025 M hydrogen peroxide for 5 h, non-viable floating seeds were removed, and seeds were then air-dried for 3 h. Mustard seeds were treated with dilute potassium permanganate solution for 20 min before planting. Basil seeds were planted without preliminary treatment per manufacturer recommendations.

#### 4.5.1. Mustard in Soil-Based Substrate

**Figure 9:**
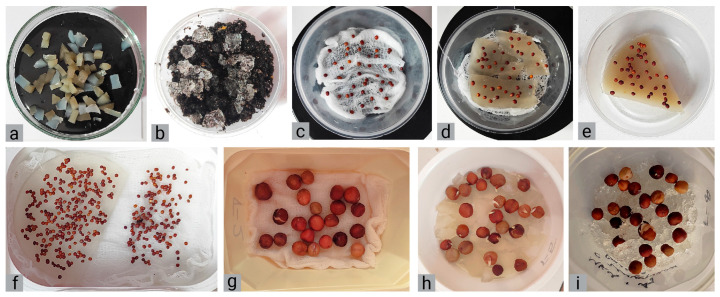
Representative photographs of hydrogel substrates and seed placement during plant cultivation experiments. (**a**) Cellulose hydrogel HG-WP cut into fragments for incorporation into soil substrate before mixing; (**b**) polyacrylate hydrogel HG-PA mixed with soil substrate; mustard seeds placed on soilless substrates at the beginning of the experiment: (**c**) gauze substrate (G), (**d**) gauze–hydrogel substrate (G-HG-WP), (**e**) cellulose hydrogel substrate (HG-WP); (**f**) dense mustard planting on gauze–hydrogel substrate (**left**) and gauze substrate (**right**); pea seeds cultivated on soilless substrates on day 2 of the experiment: (**g**) gauze substrate (G), (**h**) cellulose hydrogel substrate (HG-WP), and (**i**) polyacrylate hydrogel substrate (HG-PA).

Plastic pots (5 cm diameter) were filled with 15 g of prepared soil. To each pot, 3 g of swollen hydrogel cut into approximately 5 × 5 mm pieces was added and uniformly mixed with soil, after which 10 mustard seeds were planted at 8–10 mm depth. Two watering regimes were used: daily watering (normal conditions) and irregular watering every 3 days (water stress). Controls consisted of soil without hydrogel addition. Three hydrogel types were tested: HG-WP, HG-MCC, and HG-PA (Figure 9a,b).

#### 4.5.2. Mustard in Soilless Substrate

As an alternative to soil, three soilless substrates were used: cotton gauze (G), hydrogel HG-WP itself, and their combination (G-HG-WP) (Figure 9c–e). In the combined G-HG-WP substrates, gauze was placed underneath the hydrogel layer to provide additional moisture retention. Pre-moistened substrates (2 mL water) received 40 mustard seeds placed on a 4 × 4 cm surface, corresponding to a planting density of 2.5 seeds/cm^2^. Seeds were placed on the hydrogel surface without embedding. In a second series, higher planting density (5 seeds/cm^2^) was used on G-HG-WP-d and G-control-d substrates (6 × 6 cm) (Figure 9f).

To reduce drying during the initial stages of cultivation, containers with soilless substrates were loosely covered with plastic lids during the first 3 days. After this period, the lids were removed because the roots had already penetrated into the hydrogel matrix.

#### 4.5.3. Pea in Soilless Substrate

Pea seeds were cultivated on soilless substrates consisting of gauze (control, G), cellulose hydrogel HG-WP, or polyacrylate hydrogel HG-PA (Figure 9g–i). The experiments were carried out in plastic containers with a diameter of 9 cm. In the control samples, seeds were placed directly on the pre-moistened gauze substrate. Hydrogel substrates were cut into fragments of approximately 15 × 15 mm, after which the seeds were uniformly mixed with the hydrogel pieces. Each container received 23 pea seeds.

#### 4.5.4. Basil in Soil-Based Substrate

Basil was cultivated in soil substrates containing 20 wt.% cellulose hydrogel HG-WP relative to dry soil mass. For each experimental container, 360 g of soil and 72 g of swollen hydrogel were used. The control samples consisted of soil without hydrogel addition. The experiments were carried out in plastic containers of approximately 15 × 30 cm with a depth of 15 cm. A total of 140 basil seeds were planted in rows at a depth of approximately 0.5 cm.

### 4.6. Plant Growth Conditions

All experimental series were conducted simultaneously under identical laboratory conditions at 21–25 °C and 45–55% relative humidity under natural sunlight through a window during the period from 29 of May to 29 of June 2025.

Mustard and pea plants were watered daily with 2 mL of water per sample, except for water stress experiments, in which watering was performed once every three days. Basil plants were cultivated under regular watering conditions once every three days.

Pea and mustard experiments lasted 9 and 10 days, respectively, after which harvesting was performed. Basil cultivation experiments were conducted for 30 days.

### 4.7. Evaluation of Plant Growth Parameters

For mustard cultivated in soil substrate, shoot length was measured on days 4 and 10 of cultivation. The number of surviving seedlings was recorded on day 10. After harvesting on day 10, mustard plants cultivated in soil substrates were carefully separated from the soil, and roots were cleaned using a soft brush. Above-ground biomass (shoots) and below-ground biomass (roots) were weighed separately. Shoot and root lengths were measured, and average values were calculated.

For pea experiments, germination dynamics were monitored daily during the first 5 days of cultivation by recording the number of germinated seeds on each substrate. The cumulative percentage of germinated seeds (*P*_cum_) was calculated according to Equation (6)

(6) $$P_{cum,i}\;\left(\%\right)=\frac{\sum N_{j}}{N_{total}}\cdot 100,$$

where *N_j_* is the number of seeds germinated on day *j*, and *N_total_* is the total number of seeds planted in each experimental group.

For mustard cultivated on soilless substrates, the number of germinated seeds was recorded on days 1, 3, and 5 of cultivation. Germination dynamics during the first 5 days were evaluated by calculating the cumulative percentage of germinated seeds according to Equation (6). After harvesting on day 10, plant biomass was determined separately for shoots and roots. Plant survival rate (S, %) was assessed on day 10 according to Equation (7)

(7) $$S\;(\%)=\frac{n}{N}\cdot 100,$$

where *n* is the number of living plants at harvest, and *N* is the total number of planted seeds.

For basil experiments, total plant survival and biomass per plant were evaluated. After 30 days of cultivation, plants were carefully removed from the soil. Root biomass could not be determined because the root systems became strongly intertwined during growth in the common container. Therefore, shoots were cut at the soil surface, and the total shoot biomass of all surviving plants in each container was measured collectively. The average shoot biomass per plant (*M*_avg_) was calculated according to Equation (8)

(8) $$M_{avg}=\frac{M_{total}}{n},$$

where *M*_total_ is the total shoot biomass of all surviving plants in the container and n is the number of surviving plants.

Survival rate (*S*) on the 30th day was calculated according to Equation (7).

### 4.8. Statistical Analysis

Statistical analysis and data visualization were performed using Python 3.9 with the Pandas (1.5.2), SciPy (1.10.0), Statsmodels (0.13.5), Seaborn (0.12.2), and Matplotlib (3.6.2) libraries. All experiments in this study were performed in triplicate unless otherwise stated.

Normality of data distribution was evaluated using the Shapiro–Wilk test, and homogeneity of variances was assessed using Levene’s test. For datasets with normal distribution and homogeneous variances, statistical differences between more than two groups were analyzed using one-way analysis of variance (ANOVA) followed by Tukey’s post hoc test at a significance level of *p* < 0.05. Comparisons between two groups were performed using Student’s *t*-test.

For datasets that did not meet normality assumptions or for experiments with limited replicate numbers, nonparametric analysis was performed using the Kruskal–Wallis test.

## Figures and Tables

**Table 1:** Physicochemical properties of raw materials and prepared hydrogels.

Sample	Solid Content, %	Lignin, %	Ash Content, %	Fiber Length, mm	Fiber Width, µm
WP	94.3 ± 1.1	14.2 ± 1.1	9.30 ± 0.30	1.60 ± 0.10	25.2 ± 2.1
MCC	92.1 ± 1.3	0.5 ± 0.1	0.25 ± 0.03	0.13 ± 0.01	29.2 ± 3.2
HG-WP	1.0 ± 0.1	8.2 ± 0.9	6.20 ± 0.20	0.21 ± 0.01	35.2 ± 2.3
HG-MCC	2.5 ± 0.2	0.2 ± 0.1	0.10 ± 0.03	0.15 ± 0.01	36.4 ± 1.5
HG-PA	1.5 ± 0.3	-	-	-	-

**Table 2:** Composition, preparation method, and swelling properties of hydrogels.

Code	Raw Material and Preparation Method	Concentration, wt.%	Equilibrium Water Capacity, EWC, g/g	Re-Swelling Efficiency, R, %
HG-WP	Dissolution of waste paper fibers in DMAc/LiCl	1.0	30.57 ± 1.04	0
HG-MCC	Dissolution of cotton MCC in NaOH	2.5	3.66 ± 0.04	16
HG-PA	Commercial polyacrylate salt hydrogel	1.5	50.13 ± 0.19	100

## Data Availability

The raw data supporting the conclusions of this article will be made available by the authors on request.
